# Optimal environmental and culture conditions allow the *in vitro* coexistence of *Pseudomonas aeruginosa* and *Staphylococcus aureus* in stable biofilms

**DOI:** 10.1038/s41598-019-52726-0

**Published:** 2019-11-08

**Authors:** Maria del Mar Cendra, Núria Blanco-Cabra, Lucas Pedraz, Eduard Torrents

**Affiliations:** Bacterial Infections and Antimicrobial Therapies Group, Institute for Bioengineering of Catalonia (IBEC), The Barcelona Institute of Science and Technology, Baldiri Reixac 15-21, 08028 Barcelona, Spain

**Keywords:** Applied microbiology, Biofilms

## Abstract

The coexistence between species that occurs in some infections remains hard to achieve *in vitro* since bacterial fitness differences eventually lead to a single organism dominating the mixed culture. *Pseudomonas aeruginosa* and *Staphylococcus aureus* are major pathogens found growing together in biofilms in disease-affected lungs or wounds. Herein, we tested and analyzed different culture media, additives and environmental conditions to support *P. aeruginosa* and *S. aureus* coexistence *in vitro*. We have unraveled the potential of DMEM to support the growth of these two organisms in mature cocultured biofilms (three days old) in an environment that dampens the pH rise. Our conditions use equal initial inoculation ratios of both strains and allow the stable formation of separate *S. aureus* microcolonies that grow embedded in a *P. aeruginosa* biofilm, as well as *S. aureus* biofilm overgrowth when bovine serum albumin is added to the system. Remarkably, we also found that *S. aureus* survival is strictly dependent on a well-characterized phenomenon of oxygen stratification present in the coculture biofilm. An analysis of differential tolerance to gentamicin and ciprofloxacin treatment, depending on whether *P. aeruginosa* and *S. aureus* were growing in mono- or coculture biofilms, was used to validate our *in vitro* coculture conditions.

## Introduction

Most chronic infections occur due to the inherent capacity of some bacterial pathogens to grow in biofilms^1^. Although they have been historically investigated as monoculture events, some infection-associated biofilms are currently recognized to be mainly polymicrobial and involve synergistic interactions that often worsen the disease outcome^2,3^.

Polymicrobial biofilms can develop greater antimicrobial resistance than single-species biofilms^4^. The way bacteria are distributed and interact with each other or with the host fluctuates depending on the environment^5,6^. For instance, *Pseudomonas aeruginosa*, in addition to forming large mushroom-shaped biofilm structures *in vitro*, can behave differently and form different bacterial arrangements *in vivo*. Clusters or aggregates perfectly arranged and determined by the different bacteria that are able to grow concurrently are more likely to occur during the establishment and persistence of the infection. Therefore, there is an intrinsic effect of the surrounding environment on the way microbes behave and establish their connections^3^.

Regarding human pathogenesis, cystic fibrosis (CF) is a model example of how bacterial interactions within biofilms can modulate the outcome of the disease, thus playing a critical role in the patient’s wellbeing^7^. CF is a lethal genetic disease characterized by the production of abnormal secretions in different organs^8^. Lungs especially are affected by CF. In the lung, a thick and dense mucus builds up over the pulmonary epithelium, converting it into the perfect niche for bacterial colonization and growth^9,10^. CF-affected lungs contain changing gradients of oxygen, nutrients and pH, which together provide a heterogeneous environment that favors the coexistence and proliferation of a wide range of microbial species and, consequently, exacerbating the progression of the disease^9^. *P. aeruginosa* and *Staphylococcus aureus* are two major pathogens commonly isolated from CF-affected airways and sputum. Although *S. aureus* usually colonizes the lung epithelium during childhood and *P. aeruginosa* is more likely to be acquired in the transition to adult life, both microorganisms have been detected coexisting and synergistically contributing to the disease severity^11,12^. A similar scenario is found in infected wounds, where both bacterial species can often be found infecting simultaneously^13^. Despite the knowledge that both organisms can grow in unison *in vivo*, it remains difficult to mimic the conditions that sustain this close relationship *in vitro*.

The discovery of the culture conditions able to maintain mixed *P. aeruginosa* and *S. aureus* simultaneous growth *in vitro* has become a scientific hotspot, and several studies can be found in the bibliography addressing the interactions of these microorganisms. In their attempt to elucidate the principles of the coexistence of these species, some studies have used a higher inoculation ratio of *S. aureus vs P. aeruginosa* to establish the mixed biofilm or introduced the latter once the *S. aureus* biofilm has been established^14^. Equal inoculation ratios of both microorganisms have also been tested; however, coexistence under these conditions did not last longer than 24 h or only information related to the mixed biofilm biomass (not from each organism independently within the cocultured biofilm) can be found in the literature^15–17^. Some researchers used *P. aeruginosa* supernatant to evaluate its impact on the coculture system^18–20^, while other studies were based on wound models^21,22^.

Our study focused on deciphering the optimal coculture conditions and environmental requisites that would allow the simultaneous and stable growth of *P. aeruginosa* and *S. aureus* in mixed biofilms over time. We reasoned that the achievement of a stable *in vitro* coculture biofilm, able to grow with balanced populations of both organisms and to remain for an extended period of time, would be useful to understand the pathophysiology associated with the interaction of these two-species and for generating optimized chemotherapies to treat such biofilm-related diseases. Therefore, we developed a combination of coculture conditions that enable the stable formation of *P. aeruginosa* and *S. aureus* mixed biofilms on different abiotic surfaces. The coculture biofilms were formed using equal initial bacterial inoculation ratios and grew stably for up to three days of testing in an environmental background that dampens the pH rise. Furthermore, we provide evidence that *S. aureus* survival during coculture biofilm growth with *P. aeruginosa* is strictly dependent on oxygen diffusion. To validate the combination of the coculture conditions and environmental prerequisites identified, we treated the mixed biofilms with known antibiotics to confirm differences in antibiotic tolerance depending on whether the strains were growing in mono- or coculture biofilms.

## Results

### Testing and analyzing *P. aeruginosa* and *S. aureus* balanced population co-growth in different culture media

**Figure 1:**
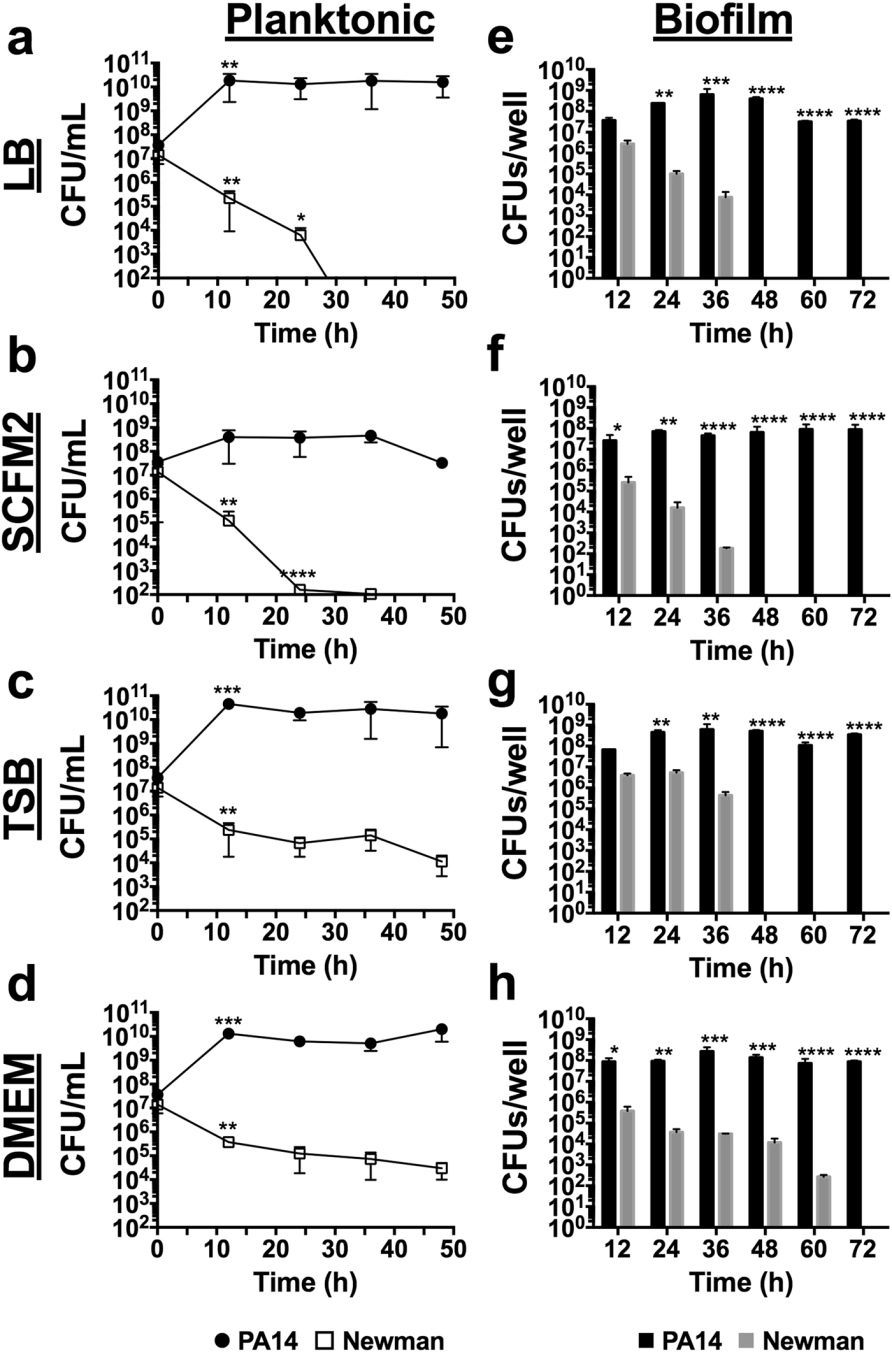
Time-course planktonic and biofilm growth of *P. aeruginosa* PA14 and *S. aureus* Newman in LB, SCFM2, TSB and DMEM. (**a-d**) log_10_ CFUs/mL of each bacterial strain during planktonic growth in coculture at the time of initial inoculation and after 12, 24, 36 and 48 h of incubation at 37 °C with shaking. (**e-h**) log_10_ CFUs/well of PA14 and Newman strains during coculture biofilm growth on 96-well polystyrene plates over 72 h of static incubation at 37 °C. Three independent experiments were performed for both experiments, and error bars indicate the standard error of the mean from the representative triplicate. Statistical significance between log_10_ CFUs/mL and between log_10_ CFUs/well at the different time points is indicated with an asterisk (*p < 0.05; **p < 0.01; ***p < 0.001*;* and ****p < 0.0001).

The culturing conditions able to block the antagonistic relationship between *P. aeruginosa* and *S. aureus in vitro* have yet to be discovered. As shown in Fig. 1a-LB, when both bacterial strains are grown together in Luria-Bertani (LB) medium, *P. aeruginosa* tends to dominate the culture and compromise *S. aureus* survival in the system (at 28 h). As our goal was to achieve balanced and stable *P. aeruginosa* and *S. aureus* mixed growth, selection of the bacterial strains to use was thought to be crucial to accomplish our objective. In this study, we used the *P. aeruginosa* PA14 strain together with the *S. aureus* Newman strain. This pair of microorganisms has been used in other polymicrobial studies^15,18,23,24^; hence, we considered them suitable for use in our study.

LB, tryptic soy broth (TSB), Dulbecco’s modified Eagle’s medium (DMEM) and synthetic cystic fibrosis sputum medium 2 (SCFM2) were tested as a base to develop a medium that maintains the coexistence and growth of both organisms over time. Thus, mixed cultures of PA14 and Newman strains and the respective CFUs were analyzed after 0, 12, 24, 36 and 48 h of incubation. For PA14-Newman planktonic cultures grown in LB and SCFM2, a significant decrease in *S. aureus* Newman CFUs/mL (*p* < 0.05) was detected during the initial 24 h of incubation with no CFUs counted after 24–36 h post-initial inoculation (Fig. 1a,b - LB and SCFM2). In contrast, TSB and DMEM maintained *S. aureus* Newman survival at ~10^5^ CFUs/mL over the 48 h with no significant changes detected after 12 h (*p* > 0.05; Fig. 1c,d - TSB and DMEM). PA14 growth was stable and maintained at ~10^10^–10^11^ CFUs/mL in the different media tested except for SCFM2, in which the strain reached maximal growth at ~10^9^ CFUs/mL (Fig. 1 - planktonic).

Since *P. aeruginosa* and *S. aureus* are commonly found in chronic infections promoted by biofilm formation^11^, this type of growth was examined next. Coculture biofilm growth was assessed in independent 96-well polystyrene microtiter plates after 12, 24, 36, 48, 60 and 72 h (Fig. 1 - biofilm). Mixed biofilms grown in LB and SCFM2 showed similar patterns for PA14 and Newman strains in planktonic growth. However, while *Pseudomonas* maintained a constant number of biofilm-forming CFUs during the 72 h checked, the presence of Newman in the mixed biofilm progressively decreased, and by 48 h no *S. aureus* CFUs were detected in the cocultured biofilm (Fig. 1e,f - LB and SCFM2). Similar to the results obtained in the planktonic experiments, coculture growth in TSB or DMEM enhanced *S. aureus* survival in the mixed biofilm. However, while TSB supported Newman growth at ~10^6^ CFUs/well for 36 h of incubation, *S. aureus* survival dropped by 48 h, with no countable CFUs detected from that time onward (Fig. 1g - TSB). In contrast, DMEM promoted constant Newman growth (~10^5^–10^4^ CFUs/well) in the mixed biofilm during the initial 48 h analyzed, with a slight decrease to ~10^2^ CFUs/well after 60 h of incubation; no countable CFUs were detected only after 72 h (Fig. 1h - DMEM). Although significantly increased viability of *S. aureus* was detected when coculture biofilm was growing in DMEM, percentage numbers of the organism within the mixed biofilm did not vary among the media used (Supplementary Table S1). PA14 levels within the mixed biofilm were similar among the four media tested, with ~10^7^–10^8^ CFUs counted per well during the 72 h of the course of the experiment (Fig. 1 - biofilm).

**Figure 2:**
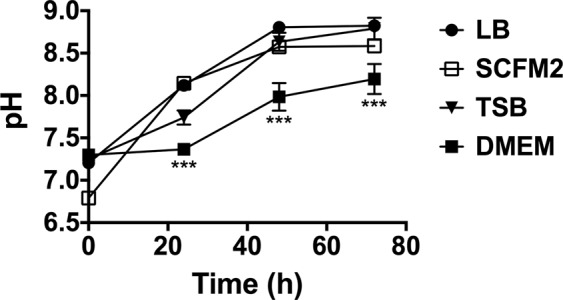
pH evolution during coculture biofilm growth in different culture media. The plot shows the pH measurements of the different supernatant phases of *P. aeruginosa* PA14 and *S. aureus* Newman coculture biofilms grown in LB, TSB, SCFM2 and DMEM. Measurements were performed at the time of initial inoculation (0 h) and after 24, 48 and 72 h of incubation at 37 °C. Statistical significance of the pH measured in DMEM at different time points compared to the pH measured in the other media at the same time points is denoted with asterisks (***p < 0.001).

The physiochemical environment where microbes grow can influence the bacterial distribution in the community^3^. Furthermore, pH homeostasis is critical to maintaining the integrity of all living cells^25,26^. Therefore, we next thought it important to examine the pH change during the coculture biofilm growth in the different media tested. The pH was evaluated at the initial establishment of the biofilms in microtiter plates at 37 °C and after 24, 48 and 72 h. Differences in pH changes were found depending on if biofilms were grown in monoculture (Supplementary Fig. S1) or coculture (Fig. 2). As shown in Fig. 2, the pH rapidly increased when biofilms were grown in LB, SCFM2 or TSB, reaching pH ∼8.7–8.8 by 48 h of incubation when growing in coculture. However, in DMEM, the pH was reduced and measured ∼0.5–1 lower, (*p* < 0.001) compared to that of the other media during the 72 h tested. This rapid increase of pH was not observed when both organisms were grown in monoculture biofilms (Supplementary Fig. S1). In that case, pH tended to be maintained or acidified (especially for *S. aureus* monoculture biofilms), during the 72 h examined.

Taken together, these results indicate that DMEM has the highest potential to maintain *S. aureus* survival over time when grown together with *P. aeruginosa*.

### Optimizing *P. aeruginosa* and *S. aureus* coculture conditions

Several conditions have been described to influence *P. aeruginosa* and *S. aureus* fitness. Accordingly, we next looked for molecules or additives to supplement the DMEM with the aim of increasing Newman survival in the mixed biofilm. Therefore, nicotinamide adenine dinucleotide phosphate (NADPH; 0.2 mM), adenosine monophosphate (AMP; 10 mM), bovine serum albumin (BSA; 5% w/v) and L-arginine (L-arg; 0.4% w/v) were chosen to be tested for the ability to compromise *P. aeruginosa* pathogenesis (AMP, BSA and L-arg), influence *S. aureus* fitness (BSA) and combat oxidative stress (NADPH)^16,21,27–29^.

**Figure 3:**
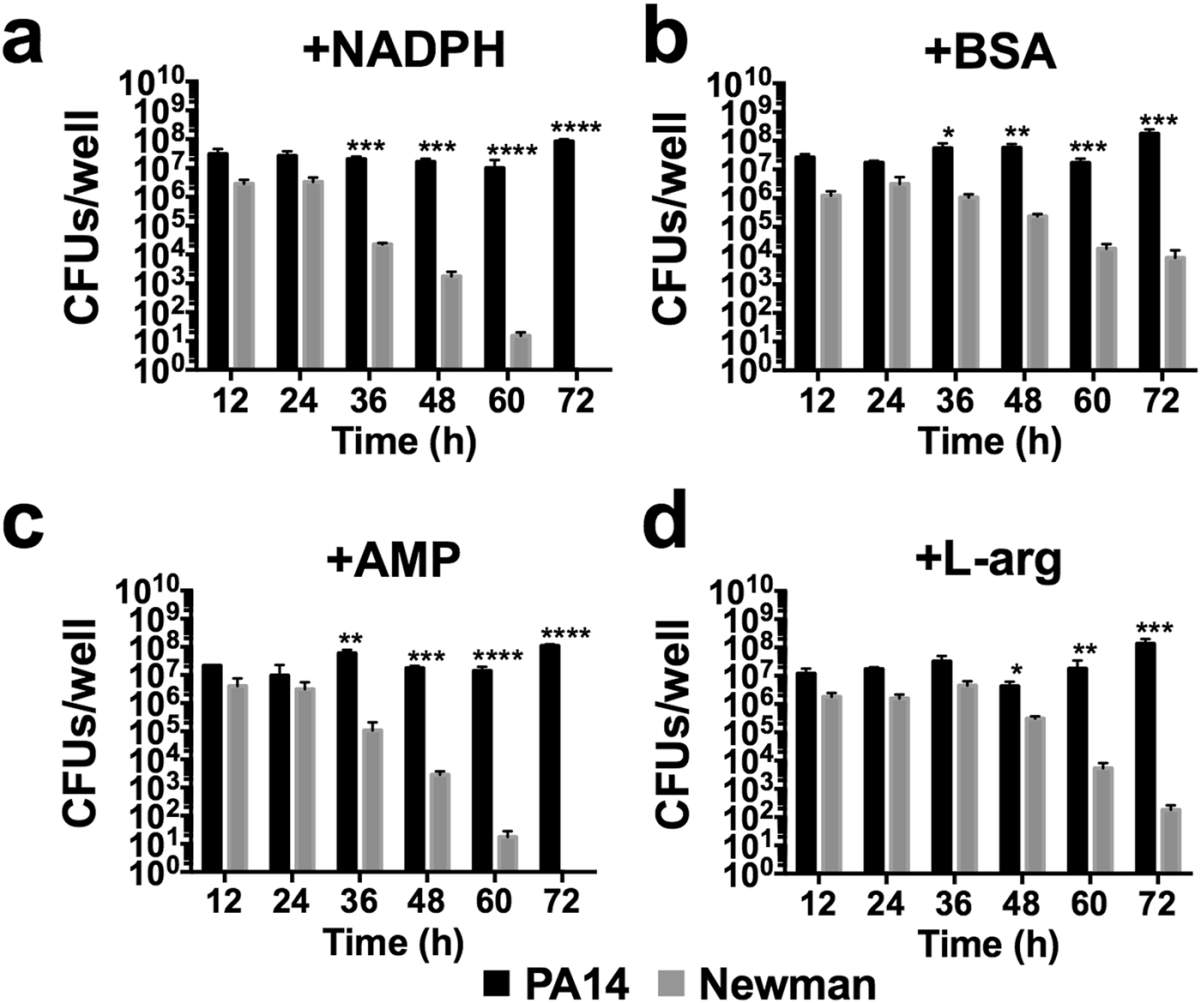
Effect of NADPH, BSA, AMP and L-arg on extending *S. aureus* survival during coculture biofilm growth with *P. aeruginosa*. Coculture biofilms were grown *in vitro* on 96-well polystyrene plates for 72 h in static conditions at 37 °C. The different graphs show biofilm cells of *P. aeruginosa* and *S. aureus* log_10_ CFUs/well during biofilm growth in DMEM supplemented with NADPH at 0.2 mM (**a**), BSA at 5% w/v (**b**), AMP at 10 mM (**c**) and L-arg at 0.4% w/v (**d**) after 12, 24, 36, 48, 60 and 72 h. Conditions were tested in triplicate, and bars represent the mean of three independent experiments. The standard error of the mean is included in the plots. Significant differences between PA14 and Newman CFUs/well at the different incubation times are indicated by asterisks (*p < 0.05; **p < 0.01; ***p < 0.001*;* and ****p < 0.0001).

To evaluate the effect of the different additives, mixed biofilms were grown in independent microtiter plates in DMEM supplemented with NADPH, BSA, AMP or L-arg (Fig. 3 and Supplementary Table S2) for 72 h at 37 °C, and the respective biofilm-forming CFUs were counted on selective agar plates. Generally, no major differences were detected in PA14 growth (CFU/well) within the coculture biofilm with any of the additives used compared to nonsupplemented DMEM (Fig. 1h - DMEM). Furthermore, *P. aeruginosa* CFUs/well were ~10^7^–10^8^ at all time-points and conditions checked (Fig. 3). A different scenario was observed with the *S. aureus* strain. Although all additives tended to maintain stable *S. aureus* Newman levels of ~10^6^–10^7^ CFUs/well during the initial 24 h (Fig. 3), the NADPH- or AMP-supplemented medium (Fig. 3a,c) did not enhance *S. aureus* growth after 36–48 h of incubation compared to nonsupplemented medium and by 60 h, no Newman CFUs were counted within the mixed biofilm in these two incubatory conditions. In contrast, coculture incubation in DMEM + BSA or DMEM + L-arg promoted sustained *S*. aureus CFU numbers of ~10^5^–10^6^ during the initial 48 h of the experiment, and these numbers decreased only after 60 h of incubation. Significantly, while in the presence of BSA, *S. aureus* CFUs were ~10^4^ CFUs/well after 72 h of incubation; the L-arg condition showed a gradual decrease in the viability of the cocci, with ~10^2^ CFUs/well counted at the end of the experiment (Fig. 3b,d). Remarkably, all additives increased the *S. aureus* cell percentage within the mixed biofilm if compared to that calculated in coculture biofilms grown in unsupplemented DMEM (Supplementary Table S1), with percentages calculated ~11–25% of the total mixed biofilm (Supplementary Table S2).

### Oxygen diffusion within PA14-Newman mixed biofilms plays a key role in maintaining balanced bacterial populations

The next step was to evaluate other environmental parameters that could influence the coculture biofilm during *in vitro* growth. Since oxygen competition between *P. aeruginosa* and *S. aureus* has been described to compromise the viability of the latter in a CF model^15^, this parameter was next assessed in our system.

**Figure 4:**
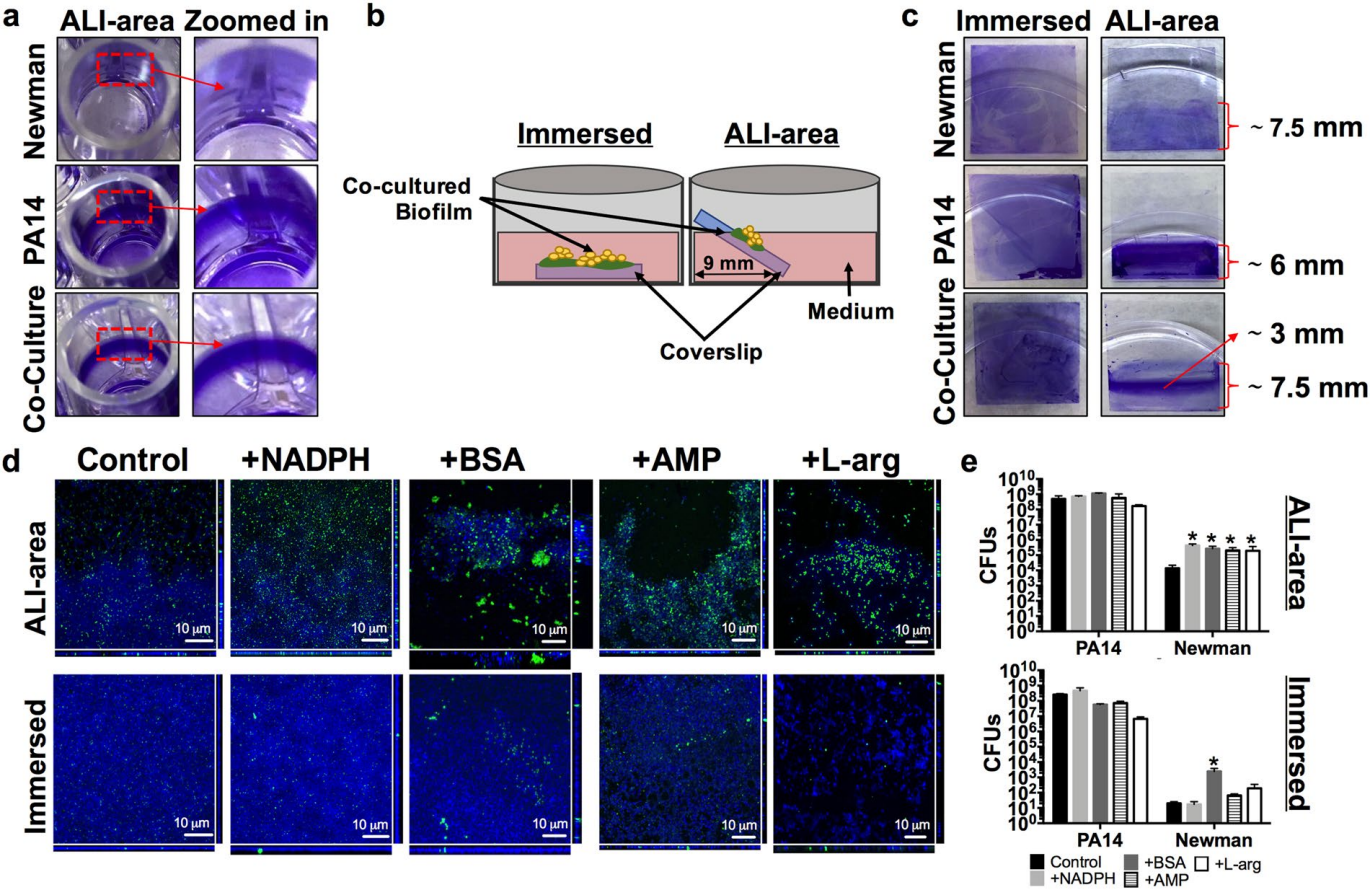
Balanced *P. aeruginosa* PA14 and *S. aureus* Newman populations growing in a coculture biofilm is dependent on the air availability. Total PA14 and Newman biomass grown in monoculture or coculture biofilms and stained with crystal violet. (**a**) Pictures show the biofilm growth of monoculture and coculture biofilms in the air-liquid interphase area (ALI area) of a 96-well plate. A zoomed image highlighting a region of interest of each biofilm is included in the figure. (**b**) Schematic representation of the coverslip position within the well during incubation of the PA14-Newman coculture biofilm immersed in the medium or in the ALI area. (**c**) Biofilm biomass staining of PA14 and Newman mono- and coculture biofilm growth over a coverslip immersed or in the ALI area. The length of the stained area in the coverslip is included on the plot. (**d**,**e**) PA14-Newman coculture biofilms grown in the ALI area or immersed in DMEM (control) and DMEM + NADPH, BSA, AMP and L-arg. (**d**) Confocal images of the mixed biofilms at the different time points were taken at 63X magnification, PA14 is shown in blue (DAPI) and Newman in green (CF^TM^-488A). A representative image of each biofilm from three independent experiments. (**e**) Plots show the different log_10_ value of PA14 and Newman CFUs covering each coverslip after 3 days of incubation. The results show the mean of three independent experiments with the corresponding standard error of the mean. Statistical significance (*p* < 0.05) compared to the relative bacterial control condition is indicated by an asterisk (*) over each bar.

We next aimed to identify the position inside the well where PA14-Newman biofilm growth occurred and to detect possible differences when compared to monoculture biofilm growth. Crystal violet staining of 48 h-old biofilms confirmed that the bacterial biofilm growth was limited to the air-liquid interphase (ALI) area of the microplate well (Fig. 4a). These results indicate that the CFU values determined in Fig. 3 come from cocultured biofilms formed in the ALI area of the microtiter plate. OD_570_ measurements revealed that PA14 forms a larger monoculture than the *S. aureus* strain. Interestingly, no additive effect was observed in the PA14 biofilm during coculture biofilm growth with *S. aureus* (Supplementary Fig. S2).

To further investigate the role of oxygen during coculture biofilm formation, we changed the *in vitro* microplate model to use coverslips. Thus, to analyze the immersed biofilm growth, the coverslip was placed in the bottom of the well of a 6-well plate and completely submerged in the medium. To assess the biofilm growing in the ALI area, the coverslip was positioned to line the well wall (see schematic representation in Fig. 4b). Crystal violet staining of 48 h-old biofilms revealed no significant differences between immersed biofilms, with similar intense violet staining detected between the mono- and cocultured biofilms (Fig. 4c). However, clear differences were observed between biofilms grown in the ALI area. While the PA14 monoculture biofilm showed more intense violet staining than the Newman monoculture biofilm, greater biofilm coverage of the coverslip was observed with the *S. aureus* strain (Fig. 4c). Measurement of the stained biofilms revealed that the Newman monoculture biofilm covered ∼7.5 mm of the coverslip, in contrast to the ∼6 mm covered by the PA14 monoculture biofilm. An intensive violet-stained band of ∼3 mm was detected in the middle of a greater coverage area of ∼7.5 mm when both organisms were grown in unison (Fig. 4c).

Coculture viability was next analyzed to assess the coexistence of both organisms within the mixed biofilm depending on the proximity to the medium surface during growth. Since increased coculture biofilm was obtained in the presence of NADPH, BSA, AMP and L-arg in 96-well plates (Fig. 3), these additives were also included in the experiment. After three days of incubation, a greater presence of *S. aureus* was observed by confocal microscopy when biofilms were grown in the ALI area compared to when they were grown completely immersed in the medium (Fig. 4d). Increased growth of *S. aureus* in the ALI area was also confirmed by CFU counting (Fig. 4e). In the nonsupplemented DMEM (control) and DMEM + NADPH conditions, the *S. aureus* strain appeared to be dispersed over the glass, whereas in DMEM + BSA, DMEM + AMP and DMEM + L-arg, the strain emerged embedded in aggregates within the PA14 biofilm (Fig. 4d - ALI area). In the validation of the results obtained in Fig. 3, significantly increased numbers of the Newman strain were counted in the mixed biofilms incubated in DMEM including the different additives (Fig. 4d). A different scenario was observed when biofilms were grown immersed in the medium, wherein PA14 completely covered the different coverslips, and the Newman strain was barely detected (Fig. 4d - Immersed). Only Newman CFUs counted in the mixed biofilm grown in DMEM + BSA showed a significant increase relative to the control condition (*p* < 0.05), although the levels were drastically reduced compared to those counted in the ALI area (Fig. 4e). Interestingly, the addition of L-arg to the system resulted in a more dispersed PA14 biofilm formation compared to that visualized with the other additives (Fig. 4d - L-arg).

Overall, these results confirm that although different additives increase *S. aureus* survival during mixed biofilm growth with *P. aeruginosa*, biofilm formation in the ALI area is fundamental to achieving this enhanced survival of *S. aureus*.

### A gradient of dissolved oxygen in the coculture biofilm system explains the differential bacterial growth

**Figure 5:**
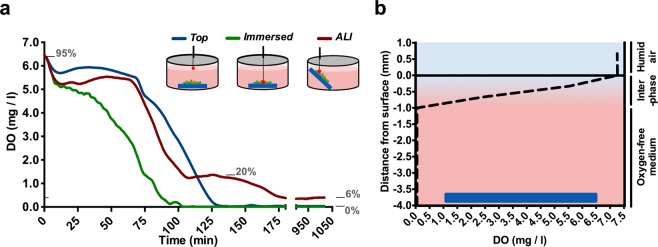
Differences in oxygen concentration depending on the position in the well where PA14 and Newman are cocultured and growing. Dissolved oxygen concentration (DO) was determined in the different areas of interest in the PA14 and Newman *in vitro* coculture biofilm model. (**a**) Plot shows the DO (mg/L; ppm) given by the optical fiber microsensor, depending on the position placed in the coculture system: top, immersed and in the ALI area. A schematic showing the approximate position of each measurement is included in the plot. The equivalence of significant DO values to the percentage of dissolved oxygen saturation (gray numbers) is provided as a reference. (**b**) DO at different depths of the culture well after the immersed area reached a DO of 0 mg/L (approximately 85 min of incubation). An interpretation of the oxygenation states across the different layers is provided on the right: **1**, humid air (the oxygen value is a result of the saturation of the sensor); **2**, oxygenated interphase; and **3**, anaerobic culture.

Different oxygen concentrations across the culture system may explain differential bacterial growth within the coculture biofilm. To validate this hypothesis, the dissolved oxygen concentration during biofilm incubation was measured in different areas of interest using an oxygen microsensor system (Fig. 5, Supplementary Fig. S3). Thus, the oxygen consumption rates in the area immediately above the immersed biofilm (ALI area) and in the medium surface (top position) were measured and analyzed over time (Fig. 5a). Additionally, a spatial oxygen profile was also measured in the medium immediately after the bottom area became depleted of oxygen (Fig. 5b). The microsensor measurements reflected a gradual decrease in the oxygen content from ∼5.5 (∼80% of the dissolved oxygen) to ∼0 mg/L in the initial ∼80 min when the PA14-Newman combined biofilm was growing completely immersed in the medium (Fig. 5a, green line). However, sensor placement in the ALI area (Fig. 5a, red line) confirmed a maintained oxygen concentration of ∼5.5 mg/L during these initial 80 min. Progressive oxygen consumption was detected after that time point, reaching ∼1 mg/L (∼20% of the dissolved oxygen) by ∼110 min from the initial incubation. Interestingly, the oxygen concentration was sustained at ∼1 mg/L for approximately ∼50 additional minutes before decreasing to ∼0.5 mg/L (∼6% of the dissolved oxygen). The oxygen levels were then maintained at ∼0.5 mg/L without reaching complete depletion. A different result was obtained when the sensor was placed close to the medium surface (top position; Fig. 5a-blue line). In this position, and comparable to the ALI area, a sustained concentration of ∼5.5 mg/L was measured during the initial 80 min of incubation. However, in this case, a linear oxygen decrease occurred immediately after that time point, reaching 0 mg/L after ∼125 min (Fig. 5a). Analysis of the oxygen stratification at different depths revealed that most of the medium in which the coculture biofilm was growing was oxygen-free. This area showed 0 mg/mL oxygen and accounted for the initial ∼3 mm of the medium depth. Only the area closest to the surface, the first ∼1 mm, revealed oxygen content and showed a progressive increase that reached ∼6.5 mg/L (∼95% of the dissolved oxygen) at the medium surface (Fig. 5b).

These results may suggest that, given the proximity to the surface, the ALI area would not reach oxygen depletion and would be able to maintain some oxygen content even though it would be a low percentage of the dissolved oxygen.

### DMEM supports *P. aeruginosa* and *S. aureus* balanced coculture biofilm growth and stabilization over time

To reinforce the potential of our developed DMEM culture conditions in forming and preserving *P. aeruginosa*-*S. aureus* mixed biofilms, we established a continuous-flow biofilm. Continuous-flow biofilms are the closest approximation known for the growth of biofilms with similar physical, chemical and biological heterogeneity as those naturally formed *in vivo*^30^.

**Figure 6:**
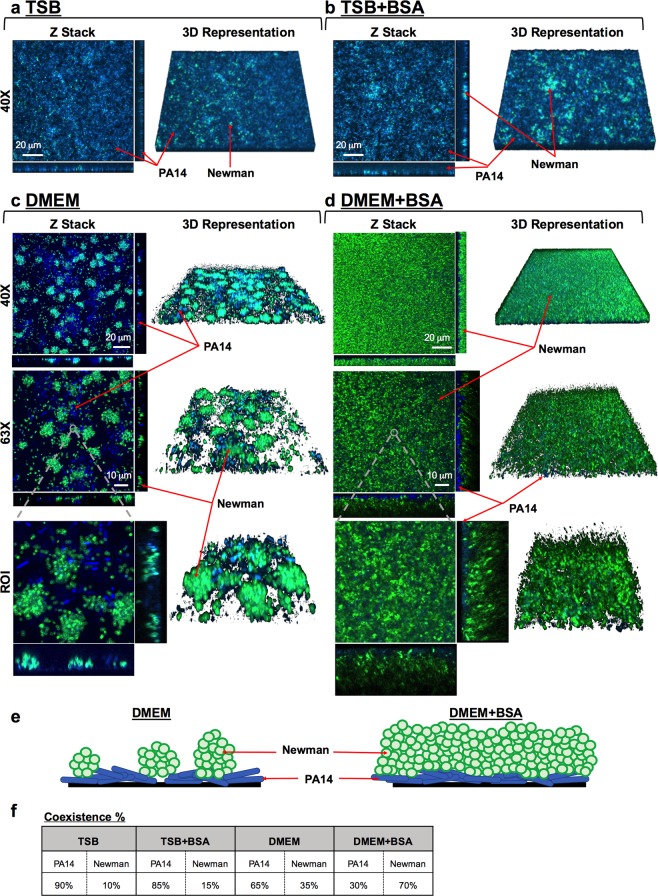
Balanced and stable *P. aeruginosa* PA14 and *S. aureus* Newman strain populations within a three-day-old mixed biofilm grown in continuous flow. PA14 and Newman were grown simultaneously in a continuous-flow biofilm in TSB (**a**), TSB + BSA (**b**), DMEM (**c**) and DMEM + BSA (**d**) for three days. At the indicated time point, the biomass growing over the different channels was stained with DAPI (blue, PA14) and CF^TM^-488A (green, Newman) and visualized using confocal microscopy. The figure shows the composite of the sum of the slices (Z Stack), with the respective orthogonal views, and the 3D representation of each mixed biofilm formed using both ImageJ and ZEN software. Mixed biofilms were tested in triplicate, and a representative image from those taken at magnifications of 40X and 63X is shown. A region of interest (ROI), including the different microscope projections and representations, is also presented in the figure. Red arrows indicate PA14 and Newman strains within the mixed biofilm. (**e**) Schematic representations of the cocultured biofilm depending on growth in DMEM or DMEM + BSA from the previous confocal microscope Z-stacks and orthogonal views. *P. aeruginosa* is represented in blue, and *S. aureus* is represented in green. (**f**) Table shows the percentage of blue *Pseudomonas* and green *Staphylococcus* present in the different cocultured biofilms calculated from an average of 5–15 different microscope stacks using the COMSTAT 2 and ImageJ software.

After three days of continuous flow, we confirmed that only DMEM was able to sustain stable growth of both populations in the biofilm. Mixed biofilms grown in TSB (Fig. 6a) or TSB supplemented with BSA (Fig. 6b) did not show any growth or increase in *S. aureus* survival within the coculture system compared to those in DMEM (Fig. 6c,d,f). Biomass evaluation using the COMSTAT 2 software determined that when the experiment was performed in TSB, *P. aeruginosa* accounted for ∼90% of the coculture biofilm, while *S. aureus* accounted for only ∼10% of the system. The addition of BSA to TSB barely increased *S. aureus* growth, and its presence in the coculture biofilm was calculated to be ∼15% in this incubatory condition (Fig. 6f). However, coculture biofilms grown in DMEM or DMEM + BSA revealed an increased presence of *S. aureus* when they were visualized with a confocal microscope (Fig. 6c,d). In general, we observed that both strains did not grow mixed together but independently and well distributed in the flow-cell channel. Interestingly, while microcolonies of *S. aureus* were detected growing embedded in the PA14 biofilm in unsupplemented DMEM (Fig. 6c), a thick and compact layer of *S. aureus* was visualized covering the *P. aeruginosa* biofilm when BSA was added to the flow system (Fig. 6d). COMSTAT 2 software revealed that this dense layer of Newman biofilm had an average thickness of ∼12.50 μm, whereas the *P. aeruginosa* biofilm growing the beneath *S. aureus* was ∼6.73 μm thick.

The orthogonal views, 3D representations and evaluation of the different regions of interest (ROIs) of the coculture biofilms were next analyzed to assess how bacterial populations were distributed within the mixed biofilm system. Our experimental approach confirmed the presence of *S. aureus* microcolonies growing in clusters within PA14 biofilms during growth with DMEM (Fig. 6c). Two schematic drawings are presented in Fig. 6e to clearly show how bacterial populations are distributed within the coculture biofilm during growth in DMEM and DMEM + BSA. Enumeration of these microcolonies revealed an average growth of approximately 65–90 of cells per bacterial aggregate, with a maximum count of approximately ∼150 of Newman cells (Supplementary Fig. S4). These image analyses also verified that *S. aureus* biofilm growth occurred on top of the *P. aeruginosa* biofilm, which was particularly evident during incubation with DMEM + BSA (Fig. 6d,e). These results are in agreement with those presented in Fig. 4. Therefore, both bacterial species maintain separate growth and distribution in coculture biofilms. Biomass evaluation revealed balanced growth of both bacterial populations of ∼35% and ∼65% for Newman and PA14, respectively, during incubation in DMEM (Fig. 6f). However, and confirming the confocal microscopy observations, a greater percentage of the *S. aureus* Newman population was measured in DMEM + BSA, accounting for ∼70% of the total coculture system (Fig. 6f).

The continuous-flow biofilm confirmed that DMEM favors the coexistence of *P. aeruginosa* and *S. aureus* in mixed biofilms. Furthermore, we also detected how the addition of BSA changed the architecture of the biofilm by increasing *S. aureus* proliferation and identified different spatial distributions of the strains in the biofilm.

### Oxygen stratification within the coculture biofilm is a key modulator of PA14 and Newman coexistence

We next wanted to verify the existence of an oxygen-stratified environment in the continuous-flow biofilm that may influence *S. aureus* survival and stable growth alongside *P. aeruginosa*. Oxygen diffusion within the biofilm was evaluated with the assumption that fresh and oxygenated medium was added continuously in the system (∼6.5 mg/mL oxygen measured with the oxygen microsensor).

**Figure 7:**
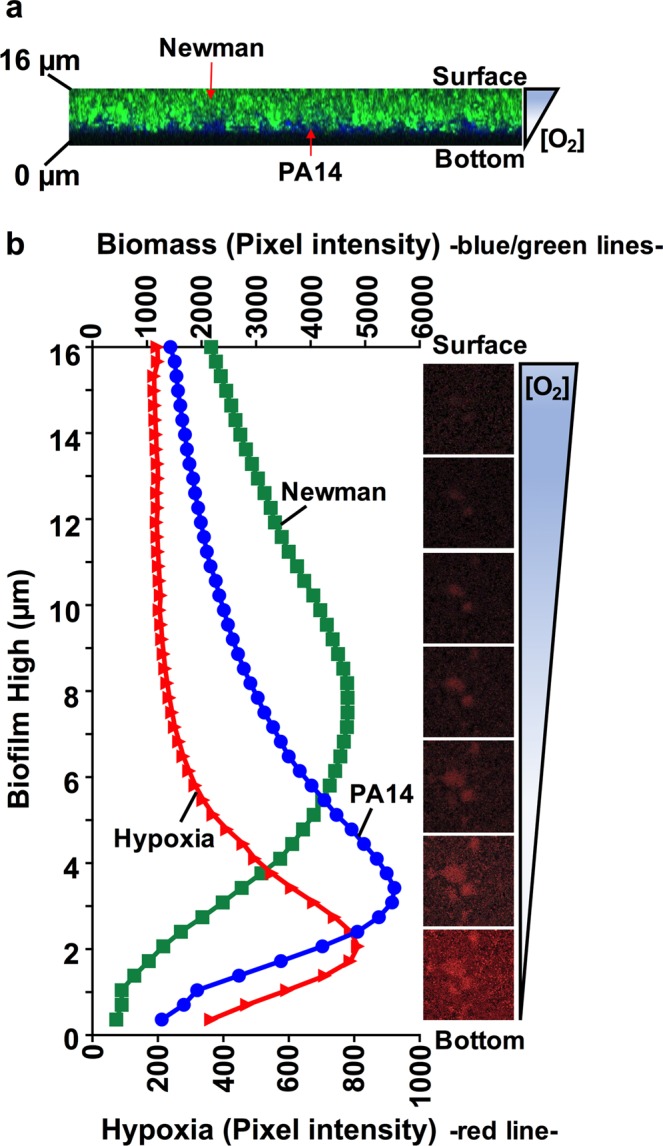
Oxygen stratification within the cocultured biofilm determines the spatial distribution of *P. aeruginosa* and *S. aureus* and their coexistence. (**a**) XZ-orthogonal view of a *P. aeruginosa* and *S. aureus* coculture biofilm grown in DMEM + BSA (see Fig. 6d). The image shows the separate position of Newman and PA14 within the ∼16 μm-biofilm and the possible oxygen gradient present in the system. (**b**) Graph shows the average pixel intensities of PA14 (blue) and Newman (Green) biomasses (plotted on the upper Y-axis) compared to the oxygen-related red intensity (plotted on the lower Y-axis) along the different thicknesses (μm) of a 3-day-old cocultured biofilm in flow. Pixel intensity averages were calculated from ten different images using ImageJ software. Included in the figure, is a sequential set of micrographs showing the red fluorescence emission given by the hypoxia probe through the different biofilm layers, indicating the oxygen stratification present along the thickness of the continuous-flow cocultured biofilm. Intense red emission relates to the hypoxic environment through the different biofilm depths (μm), from the bottom to the surface, additionally indicated with a schematic of the oxygen gradient present in the system.

Orthogonal views of the coculture biofilm grown in the flow-cell channel confirmed the clear distribution of the *S. aureus* biofilm on top of *P. aeruginosa* in the area closest to the medium surface (Fig. 7a). Consequently, we next wanted to validate the presence of an oxygen gradient that could be crucial for Newman survival in the mixed biofilm, and a key modulator of the bacterial distribution detected. To probe different oxygen concentrations within the coculture system, the continuous-flow biofilm was analyzed using the Hypoxia Probe dye. The key attribute of this dye is that its fluorescence is quenched by oxygen, so the lower the oxygen concentration is, the greater the red fluorescence signal emitted by the stained cells, thus allowing the detection of an anaerobic environment. Red-light emission (hypoxia) through the different layers of the coculture biofilm confirmed the bright fluorescence during the initial ∼4 μm of biofilm thickness that gradually decreased across the bacterial community. Red fluorescence was barely detected in the more superficial biofilm layers, thus confirming aerobic conditions in that area (Fig. 7b). Additionally, pixel intensity analysis of the hypoxic region (red) corroborated that red emission peaked around the initial ∼2 μm of the biofilm depth. Furthermore, the PA14-Newman biomass was also quantified in this continuous-flow biofilm. *P. aeruginosa* PA14 (blue) displayed a maximum peak intensity at ∼3 μm of the biofilm depth, corresponding to the more anaerobic region of the biofilm, with fluorescence extending to a thickness of ∼10 μm. *S. aureus* Newman (green) clearly showed a pixel intensity curve shifted to higher and more oxygenic biofilm layers (Fig. 7b). Green intensity measurements confirmed that *S. aureus* was barely detectable in the initial ∼3 μm of the mixed biofilm (coinciding with the anoxic part of the biofilm); the intensity increased, achieving a maximum and extended peak at ∼6–9 μm of depth, with fluorescence emission extending to ∼14–10 μm of the biofilm thickness.

Taken together, these results demonstrate that the coculture biofilm formed in continuous flow also displays oxygen stratification across the different biofilm layers. *P. aeruginosa* growth occurred across the deepest layers of the biofilm, where less oxygen content was detected, while *S. aureus* biofilm growth was quantified closer to the biofilm surface, corresponding to the most oxygenated area.

### Antibiotic resistance of *P. aeruginosa* and *S. aureus* is critically increased during coculture biofilm growth

Biofilm-associated infections have historically been treated as single-species events. Nevertheless, some of these infections are now known to be composed of multiple combinations of bacteria, involving complex interactions that can influence their fitness and antibiotic tolerance^2,4^. The demonstration of altered antibiotic susceptibilities of PA14 and Newman when grown in coculture biofilms was thought to be necessary to validate the combination of coculture conditions and environmental prerequisites identified in this study. Even though in DMEM + BSA condition presence of *S. aureus* within the mixed biofilm grown in microtiter plates was detected also after 72 h of coculture growth, we chose to evaluate the antimicrobial tolerance after 48 h since according to Fig. 3b, at that time-point Newman’s CFUs in the coculture biofilm were counted ∼100−fold higher than at 72 h. Hence, 48 h-old PA14 and Newman mono- and coculture biofilms were treated with ciprofloxacin (Cpx) and gentamicin (Gm). The respective biofilm-forming CFUs were subsequently counted on selective agar. The antibiotics and the range of concentrations used were chosen according to their reported minimal inhibitory concentrations (MICs) and their usage in treating both bacterial infections^4,31^. The experiment was performed in a 96-well microtiter plate and included BSA since it was the additive that showed the greatest potential for maintaining the stable growth of both bacterial populations *in vitro*.

**Figure 8:**
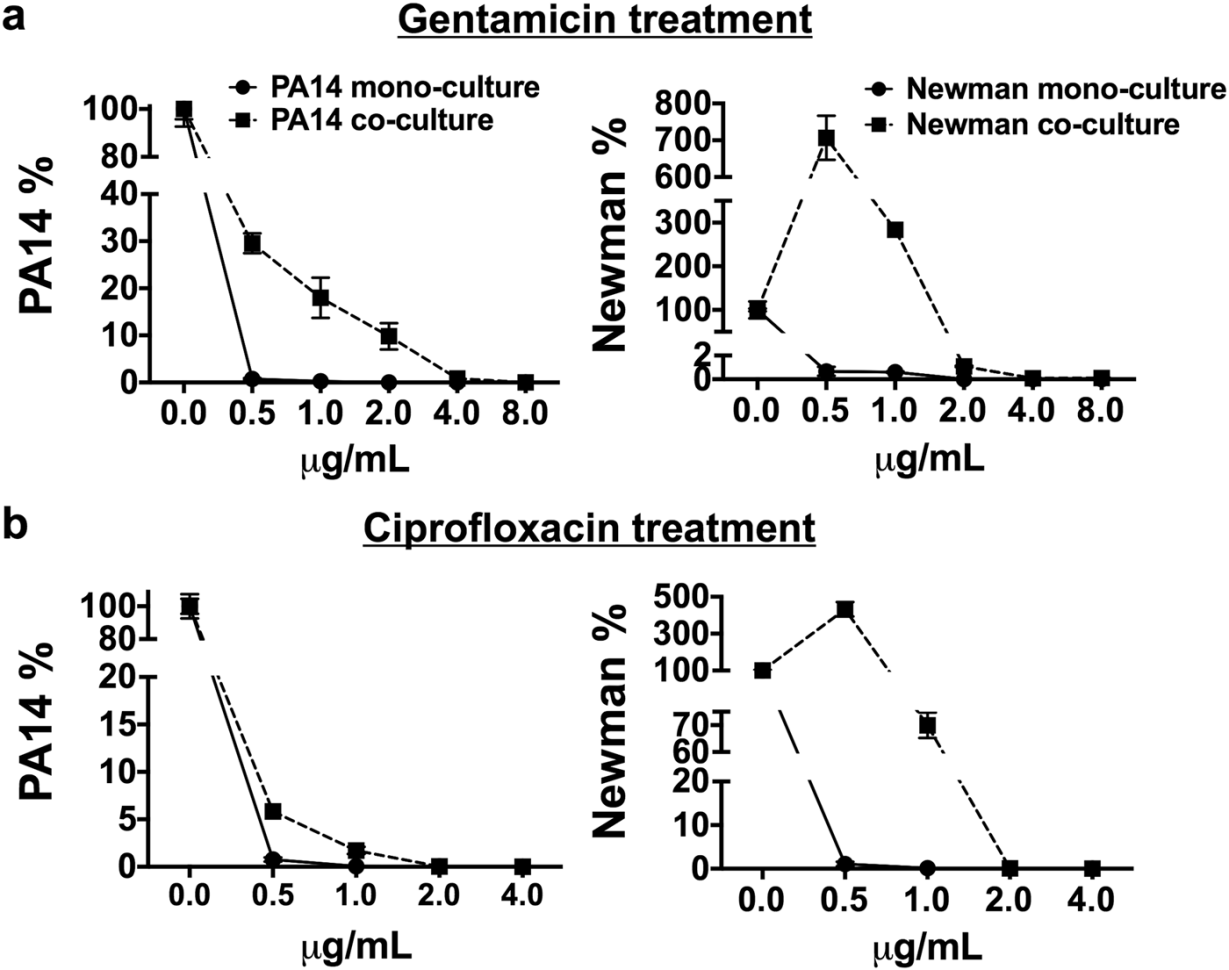
*P. aeruginosa* PA14 and *S. aureus* Newman coculture biofilms induce enhanced tolerance to antibiotic treatment compared to monoculture biofilms. After 48 h, matured mono- and cocultured PA14 and Newman biofilms grown in microtiter plates were treated with 0.5, 1.0, 2.0, 4.0 and 8.0 μg/mL gentamicin (**a**) and 0.5, 1.0, 2.0, and 4.0 μg/mL ciprofloxacin (**b**) for 15 h. Symbols in the plots represent the remaining percentage of CFUs of each strain in the biofilm after different antibiotic treatments compared to the relative untreated biofilm. Each graph shows the percentages of PA14 and Newman CFUs compared according to whether the biofilms were grown in mono- or coculture. Percentages were calculated according to the bacterial CFUs counted on selective agar plates after different antimicrobial treatments (Supplementary Table S3). Analysis of the statistical significance between the calculated percentages of bacterial CFUs that remained in the cocultured biofilm (after each antibiotic treatment) and those calculated in the monocultured biofilms revealed significance with *p* < 0.0001 in all cases.

Bacterial CFUs that remained viable within the mono- and the coculture biofilms after the antibiotic treatment were enumerated (Supplementary Table S3), and the percentages of viable CFUs that persisted in each biofilm after the different treatments, compared to each untreated biofilm, were subsequently calculated (Fig. 8). The Minimum Biofilm Eradication Concentration (MBEC) of each antibiotic against *P. aeruginosa* and *S. aureus* mono and coculture biofilms was also calculated (Table 1). Crystal violet staining of control wells confirmed that the mature biofilm grew in the ALI area of the well (data not shown). A similar behavioral pattern was observed in both organisms after the respective antibiotic treatments; however, *S. aureus* revealed a much greater benefit of growing in coculture than *P. aeruginosa*, since its viability during both treatments increased exponentially when grown in coculture with *P. aeruginosa*. As for *P. aeruginosa*, the bacterium exhibited ∼38-, ∼68- and ∼390-fold higher tolerance to Gm treatment in coculture than in monoculture biofilm growth for concentrations of 0.5, 1 and 2 μg/mL, respectively, (Fig. 8a - graph PA14%) and ∼7- and ∼27.5-fold increased to Cpx treatment used at concentrations of 0.5 and 1 μg/mL (Fig. 8b - graph PA14%). The MBEC determinations (Table 1) confirmed that Gm treatment at ≥0.5 and ≥4 μg/mL and Cpx treatment at ≥0.5 and ≥2 μg/mL cleared PA14 from the mono- and coculture biofilms, respectively (Fig. 8a,b -graph PA14%). The percentage calculations revealed that growing *S. aureus* in coculture enhanced its capacity to persist in the presence of Gm >1000-fold, at a concentration of 0.5 μg/mL, and >470-fold at 1 μg/mL (Fig. 8a - graph Newman%). A similar pattern was detected for Cpx treatment; within a coculture biofilm with *P. aeruginosa*, the viability of *Staphylococcus* increased ∼400-fold when the antibiotic was used at 0.5 μg/mL and >468-fold when it was used at 1 μg/mL (Fig. 8b - graph Newman%). Only doses ≥2 μg/mL of Gm or Cpx cleared *S. aureus* from the mixed biofilm. In contrast, treatments with 0.5 μg/mL with any of the antibiotics used was sufficient to clear *S. aureus* from a 48 h-old monoculture biofilm (Fig. 8a,b - graph Newman% and Table 1). When these high concentrations were used, the viability of the *S. aureus* strain was drastically reduced.

**Table 1:** MBEC values of gentamicin and ciprofloxacin antibiotics against *P. aeruginosa* PA14 and *S. aureus* Newman in monoculture or coculture biofilms.

	MBEC (µg/mL)	Biofilm reduction
**Gentamicin**		
PA14 monoculture	0.5	≥99%
PA14 coculture	4	≥99%
Newman monoculture	0.5	≥99%
Newman coculture	2	≥99%
**Ciprofloxacin**		
PA14 monoculture	0.5	≥99%
PA14 coculture	2	≥99%
Newman monoculture	0.5	≥99%
Newman coculture	2	≥99%

Comparisons between the percentages of the viable *P. aeruginosa* and *S. aureus* CFUs in the coculture biofilm after the antibiotic treatments to those calculated in the monoculture biofilms revealed significance in all cases (*p* < 0.001). These results confirm the increased antibiotic tolerance of *P. aeruginosa* PA14 and *S. aureus* Newman strains when grown in a mixed biofilm rather than in a monoculture biofilm, thus validating the coculture conditions established in this study.

## Discussion

Biofilm-associated infections are currently a critical worldwide threat^32–34^. The increasing emergence of antimicrobial-resistant bacteria and the knowledge that some of these infections are polymicrobial challenge the antimicrobial chemotherapy to administrate and aggravates the disease outcome^2,35,36^. *P. aeruginosa* and *S. aureus* are two major pathogens commonly found growing together in intricate biofilms in disease-affected lungs^11^ or wounds^13^. Herein, we have unraveled the potential of DMEM to sustain a *P. aeruginosa* PA14 and *S. aureus* Newman combined biofilm for up to three days *in vitro* and identified BSA as a valuable and critical additive that significantly increases *S. aureus* survival and growth in the coculture system. Remarkably, we also demonstrated the importance of continuous oxygen diffusion in limiting *S. aureus* survival and keeping the growth of both bacterial populations balanced, highly influencing their distribution in the coculture biofilm. Furthermore, using our developed coculture conditions, we confirmed that the antimicrobial susceptibilities of *P. aeruginosa* and *S. aureus* differ depending on whether they are growing in monoculture or coculture in biofilms.

Among the different media evaluated (LB, TSB and SCFM2), DMEM was the greatest at controlling *S. aureus* survival during simultaneous growth (planktonic and biofilm) with *P. aeruginosa*. DMEM is a rich culture medium used in routine cell culture experiments, which contains numerous amino acids, vitamins, and inorganic salts, among other components^37^. Remarkably, the D-glucose concentration in this medium is ∼17.5 mM, which is greater than the usual 0.2% (∼11.1 mM) added to LB or TSB medium in routinely used biofilm formation protocols^38–40^, or the 3 mM present in the SCFM2 medium^41^. In healthy people, glucose concentration in the airway surface liquid (ASL) is ∼0.4 mM, 12 times lower than blood glucose^42,43^. However, lung inflammation, caused by diseases such as CF or chronic airway inflammation, increases glucose flux through the epithelial cell membrane, raising ∼10–12 times the glucose concentration in the ASL. Different studies have described how increased glucose levels in ASL promote bacterial lung infection^42,44,45^. Furthermore, diabetes affected-people have wound healing issues and increased risk of infection due to an impaired host defence^46^. Glucose is not the preferred carbon source of *P. aeruginosa*^47^ but is the preferred carbon source of *S. aureus*, which exhibits preferential uptake of this sugar, especially during infection^48^. Therefore, it is plausible to hypothesize that *S. aureus* could benefit from the high glucose concentration in DMEM and grow without competition for the substrate. Additionally, a differential planktonic growth pattern was detected in *S. aureus* Newman depending on whether the bacterium was grown in DMEM or TSB (Supplementary Fig. S5). In DMEM, *S. aureus* grew rapidly and achieved late-exponential/stationary phase, with growth maintained during the course of the experiment. However, in TSB, the strain exhibited the usual bacterial growth curve with lag, exponential and stationary phases. This result indicates increased efficiency of *S. aureus* growth in DMEM, especially during the initial stage of growth, which we hypothesize could be beneficial during simultaneous biofilm growth with *P. aeruginosa* to rapidly form a microcolony after initial attachment, thus providing defense against *Pseudomonas*^49^. *S. aureus* growth in firmly packed microcolonies during coculture biofilm growth with *P. aeruginosa* PAO1 has been previously seen by Yang and coworkers^50^. In our study, this microcolony formation may also be facilitated by the increased concentration of NaCl present in the DMEM formulation (∼120 mM), which is >3-fold higher than in LB, TSB or SCFM2 and has been seen to stimulate biofilm aggregation and growth^51,52^. The concentration of glutamine present in DMEM (∼2.5 mM) may also affect the coexistence of both microorganisms by diminishing the specific competition for this amino acid, as a nitrogen and energy source, which has been recently reported to occur early during coculture^53^.

Significantly, DMEM contains HEPES (4-(2-hydroxyethyl)-1-piperazineethanesulfonic acid; ∼15 mM), which is considered a “good buffer” for its limited effect on biochemical reactions and for being chemically and enzymatically stable, among other properties^54^. The buffering properties of HEPES were evident in the growing coculture biofilms, and the pH rise was dampened compared to that measured in the other media (Fig. 2). pH homeostasis is critical to maintaining the integrity of cytoplasmic proteins in all living cells, and their optimal pH fluctuates in a narrow range of 7.4–7.8^25,26^. Although SCFM2 contains 3-morpholinopropanesulfonic acid, it failed to maintain the pH levels in the coculture. This synthetic CF medium was developed based on CF sputum that contained high concentrations of *P. aeruginosa*^41^, which may explain why SCFM2 did not support *S. aureus* viability in the system. Additionally, a factor increasing the perturbation of *P. aeruginosa* and *S. aureus* coexistence in this fluctuating pH environment is the production of different proteases by *P. aeruginosa* in an alkaline environment of pH ∼8. In particular, the *P. aeruginosa* staphylolytic protease LasA possesses optimal activity at approximately pH 8.5^55–57^.

DMEM supplementation with BSA and L-arginine increased *S. aureus* viability throughout the 72 h of coculture biofilm growth with *P. aeruginosa*. Although coculturing the biofilm in DMEM + L-arg increased the Newman biofilm-forming CFUs, confocal microscopy revealed an altered coculture biofilm architecture, with disaggregated clumps covering the coverslip (Fig. 4d), which is consistent with the phenotype promoted by L-arginine that has been seen in other biofilm communities^58–60^. Thus, we conclude that among the additives tested, BSA possesses the greatest potential to increase *S. aureus* viability and maintain the population balance in a well-engineered coculture biofilm with *P. aeruginosa*. Subsequent experiments in flow-cell biofilms corroborated the effect of BSA in increasing *S. aureus* survival and growth during mixed biofilm formation with *P. aeruginosa* (Fig. 6d). Although albumin has been described to diminish *P. aeruginosa* killing of *S. aureus* in wounds by binding and sequestering *Pseudomonas* quorum sensing molecules^21^, which is also likely what occurred in our model, we also believe albumin has a direct effect on *S. aureus* viability, although further experiments are needed to confirm this hypothesis. Albumin is the main plasma protein and a carrier of numerous molecules, such as metals and other ions, bilirubin, amino acids, fatty acids, enzymes, and hormones^61^. With an unknown mechanism, it is known that the presence of this protein in the culture medium enhances *S. aureus* growth exponentially, possibly by scavenging traces of protein-bound nutrients^62–64^. Therefore, we suggest that albumin could play a direct role in inducing prompt microcolony formation by increasing the proliferation rate of *S. aureus* in a hostile environment with *P. aeruginosa*. Furthermore, although expression of different virulence factors of *Pseudomonas* has detected increased during coculture growth with *S. aureus* (e.g. LasA protease or pyocyanin production)^53^, some staphylococcal factors (e.g. the Panton-Valentin leukocidin protein) have been also observed during these coculture conditions, which may be playing a role also in competing with the *Pseudomonas*^65^.

Remarkably, in this study, we demonstrate an important role for oxygen in achieving continuous and stable coculture biofilms of *P. aeruginosa* and *S. aureus*. We also observed a differential distribution of the bacterial populations depending on the oxygen content in the surrounding environment. Lungs are not entirely aerobic, especially those affected by CF, in which the thick mucus present in the airways generates diverse oxygen content between pulmonary regions, thus enhancing the heterogeneity of microbes able to proliferate and persist at the same site^66–68^. Oxygen diffusion also mediates the different spatial distribution of bacteria in wounds; hence, *P. aeruginosa* has been found deeper in the tissue than *S. aureus*, which grows predominantly at the wound surface^13^. *Pseudomonas* is able to grow in anaerobic conditions in the presence of nitrates, which are included in the DMEM formulation. *S. aureus* encodes a set of genes required for growth either aerobically or anaerobically^69,70^; however, aerobic respiration is preferred by *S. aureus* during monoculture growth. Despite these metabolic preferences, during simultaneous growth with *P. aeruginosa*, oxygen competition between organisms drives *S. aureus* to shift to fermentative metabolism. This metabolic shift is also triggered by the expression of siderophores, phenazines and other exoproducts (i.e., 2-heptyl-4-hydroxyquinoline N-oxide (HQNO) production) by *Pseudomonas* that compromise *S. aureus* viability^15,71^. In our model, increased *S. aureus* Newman survival was detected when the coculture biofilms were grown in the ALI area, either in a 96-well plate or over coverslips, rather than during complete medium immersion. Furthermore, we confirmed the existence of an oxygen gradient across the medium depth during biofilm growth, with a continuous micro-oxygenated phase at the medium surface (corresponding to the ALI area). Therefore, we hypothesize that persistent oxygen diffusion at the ALI area of the coculture system increases *S*. *aureus* survival within the mixed biofilm by diminishing the oxygen competition between the organisms and the subsequent production of *P. aeruginosa* molecules that eventually kill *S. aureus*.

The public health concern about biofilm-associated diseases is linked to the altered antimicrobial susceptibilities that these communities present^36^. Unoptimized therapies and variations in the antibiotic concentration across the biofilm layers promote the development of resistance since bacteria are usually exposed to subinhibitory concentrations of the antimicrobial^4^. Exacerbating the problem is the fact that these biofilms tend to be composed of multiple species, with different fitness values and high levels of cooperative and synergistic interactions that are often detrimental to the host^2,4^. For instance, the simple addition of *P. aeruginosa* supernatant to *S. aureus* Newman biofilms has been seen to be sufficient to increase the tolerance of *S. aureus* to a wide range of antibiotics, such as vancomycin, tobramycin and oxacillin^18^. Furthermore, it has been seen that continuous exposure of *S. aureus* to *P. aeruginosa* HQNO promotes the formation of antibiotic-resistant small colony variants of the bacterium, enhancing its resistance to aminoglycosides^23^. *P. aeruginosa* and *S. aureus* showed enhanced tolerance to gentamicin and ciprofloxacin antibiotics when they were grown in coculture biofilms (Fig. 8). Hence, we believe that the use of the conditions revealed in this study allows the stable formation of *P. aeruginosa* PA14 and *S. aureus* Newman coculture biofilms involving the intricate and interspecific relations responsible for influencing the antimicrobial tolerance of each strain.

In summary, in this study, we elucidated the potential of DMEM for *P. aeruginosa* and *S. aureus in vitro* coculture. Additionally, we have discovered that supplementing DMEM with BSA and providing continuous oxygen diffusion allows the formation of a mature mixed biofilm with stable populations of *P. aeruginosa* and *S. aureus*. This study provides useful insights about the establishment of a *P. aeruginosa* and *S. aureus* combined biofilm *in vitro*, which we believe would be of help for the study of phenotypes derived from this clinically challenging bacterial cooperation as well as for optimizing the antimicrobial therapy used to treat these infections.

## Methods

### Bacterial strains and growth conditions

*Pseudomonas aeruginosa* PA14 wild type^72^ and *Staphylococcus aureus* Newman (ATCC 13420) were used throughout this study, although *S. aureus* ATCC 12600 and ATCC 29213 were also initially tested. Overnight cultures (O/N) were performed aerobically at 37 °C in Luria-Bertani medium (LB; Scharlab, S.L., Barcelona, Spain) and in tryptic soy broth (TSB; Scharlab, S.L.) for the PA14 and Newman strains, respectively.

### *P. aeruginosa* PA14 and *S. aureus* Newman coculture medium conditions

LB, TSB, synthetic cystic fibrosis sputum medium 2 (SCFM2), prepared as previously described^41^, and Dulbecco’s modified Eagle’s medium/nutrient mixture F-12 (DMEM; Thermo Fisher Scientific, Waltham, Massachussetts) were tested. When required, reduced nicotinamide adenine dinucleotide phosphate (NADPH; 0.2 mM), adenosine monophosphate (AMP; 10 mM), bovine serum albumin (BSA; 5% w/v) and L-arginine (0.4% w/v) were added to the medium. O/N cultures of PA14 and Newman strains were washed twice with 1X phosphate-buffered saline (PBS, pH = 7.4). To prepare the initial mixed bacterial suspension, each strain was inoculated at a final optical density λ = 550 nm (OD_550_) of 0.05 for planktonic experiments, and an OD_550_ of 0.10 for biofilm experiments.

### Planktonic coculture growth

Mixed bacterial suspensions in a final volume of 20 mL were incubated aerobically at 37 °C with vigorous shaking (200 rpm). At given time points, each planktonic culture was serially diluted in 1X PBS and plated on LB agar (Scharlab, S.L.) to count *P. aeruginosa* CFUs, and tryptic soy agar (TSA; Scharlab, S.L.) supplemented with 7.5% (w/v) NaCl to selectively count *S. aureus* CFUs^73^.

### Coculture biofilm growth under static conditions

Static *P. aeruginosa* PA14-*S. aureus* Newman mixed biofilm growth was tested on (i) plastic and (ii) glass surfaces:

(i) **Plastic surface:** 200 μl of each mixed bacterial suspension was inoculated in triplicate in 96-well polystyrene plates with a flat bottom (Corning; Sigma-Aldrich, San Luis, Missouri) and incubated at 37 °C without shaking. At different time points, the planktonic phase was removed, and each well was washed three times with 1X PBS. The biofilm phase formed over the wall of each well was removed using a pipette tip, and the triplicates were mixed together. For CFU quantification, each biofilm cell suspension was placed in an ultrasonic bath (USC100T, VWR) for 5 min and subsequently vortexed for 30 seconds, to help dispersing the biofilm. Bacterial suspensions were then serially diluted in 1X PBS and plated on selective agar as described for planktonic growth. Separately, the biofilm mass formed inside the well was stained with 0.1% (w/v) crystal violet, and the biomass (OD_570_) was determined as previously described^74^.
(ii) **Glass surface:** 18 × 18 mm coverslips (Menzel-Gläser, Thermo Fisher Scientific) were placed in a 6-well polystyrene plate with lid (Dd biolab, Barcelona, Spain), and each well was filled with 3 mL of the PA14-Newman bacterial suspension. The coverslip was positioned completely immersed in the bacterial suspension or only half immersed. The half immersion, with the air-liquid interphase (ALI) area, was achieved by placing the coverslip at a ∼45° angle against the wall of the well (see Fig. 4b). Unattached cells were removed after three hours of incubation, and 3 mL of fresh medium was subsequently added again. This procedure was repeated every 12 h during the course of the experiment. At the given time points, the coverslip, covered with the established mixed biofilm, was gently washed with PBS. To determine bacterial CFUs, biofilm-forming cells were scraped off the coverslip, and serial dilutions were plated on selective agar plates as described above. In addition, the biomass of the PBS-washed coverslip was stained with crystal violet or with different dyes for confocal microscopy as described below.

### pH measurements

The pH of the supernatant phase of each PA14 and Newman monoculture and coculture biofilm was measured using a GLP 21 pH meter (Crison®, Hospitalet de Llobregat, Barcelona). For this experiment, biofilms were grown in a volume of 3 mL in 6-well polystyrene plates. pH measurements were taken in triplicate for each culture medium and time point, directly in the microplate well where the biofilm was growing.

### Confocal laser scanning microscopy and image analysis

To differentially stain *P. aeruginosa* PA14 and *S. aureus* Newman, a Bacterial Viability and Gram Stain kit (Biotium, Fremont, California) was used following the manufacturer’s instructions. This kit uses the cell membrane differences between gram-negative and gram-positive bacteria to differentially stain each species. Briefly, the kit combines DAPI to stain the bacterial DNA blue with CF^TM^-488A-wheat germ agglutinin (WGA) to bind, specifically, the N-acetylglucosamine present in the peptidoglycan of the cell wall in gram-positive bacteria. Consequently, *P. aeruginosa* PA14 will be stained blue, and *S. aureus* Newman will be stained green. To detect bacterial cells growing in different oxygen concentrations, we used the phosphorescent light-emitting iridium complex Hypoxia Probe (Organogenix; Bionova científica, Barcelona, Spain) according to the manufacturer’s instructions.

Stained bacteria were imaged using a Zeiss LSM 800 confocal laser scanning microscope (CSLM, Zeiss, Oberkochen, Germany), and images were analyzed with ImageJ and ZEN (Zeiss software).

### Assessment of the oxygen concentration in the coculture system

The coculture biofilm was established as described above but using lids for the 6-well plates with small holes drilled in the desired locations. After the addition of fresh medium, the plate was transferred to equipment for oxygen measurement described in Supplementary Fig. S3. The dissolved oxygen was measured using an Oxymicro Fiber-Optic Sensor System (World Precision Instruments) connected to a micro-optode oxygen sensor in a syringe-type housing (PreSens, Regensburg, Germany); the needle of the housing crossed the lid of the plate, and the optical fiber was placed using the plunger at the desired location. All measurements were compensated with temperature using a temperature probe placed directly above the culture plate.

Micro-optodes were calibrated using temperature-compensated measurements of water saturated with air (100% air saturation, 8.25 mg/L at 25 °C and 1 atm) and a 10 g/L solution of sodium dithionite (0% O_2_).

### Coculture biofilm incubated in a continuous-flow system

The *P. aeruginosa* and *S. aureus* mixed bacterial suspension (each strain at OD_550_ = 0.10) was inoculated in a three-channel flow-cell (DTU Systems biology, Technical University of Denmark). Media was pumped at a constant flow rate of 42 μl per minute and channel using an Ismatec ISM 943 pump (Ismatec, Wertheim, Germany), as previously described^75^. After 3 days of growth, biofilms were stained with the Bacterial Viability and Gram Stain kit or with the Hypoxia Probe and observed by confocal microscopy. Images were generated, and biomass was calculated using ImageJ and COMSTAT 2 software^75^. Percentage of *S. aureus* in the coculture biofilm was calculated taking the pixels given by *S. aureus-*CF^TM^-488A (channel 0) from the pixels given by the total DAPI staining (channel 1). *P. aeruginosa* percentage in the coculture biofilm was subsequently obtained by subtracting the percentage of *S. aureus* coverage from the total biofilm (100%).

### Antimicrobial treatments and minimum biofilm eradication concentration (MBEC) calculations

*P. aeruginosa* PA14 and *S. aureus* Newman 48 h coculture biofilms in 96-well polystyrene plates were treated with gentamicin sulfate (Panreac AppliChem, Castellar del Vallès, Spain) at concentrations of 0.5, 1, 2, 4 and 8 μg/mL and with ciprofloxacin hydrochloride (Cayman Chemical, Ann Arbor, Michigan) at 0.5, 1, 2 and 4 μg/mL to investigate possible alterations on antibiotic tolerance depending if they were cultured in mono or in coculture biofilms. Furthermore, different MBEC were also calculated according to the treatment concentration that eradicated ≥99% of the biofilms^76^. After 15 h of incubation with antibiotics, biofilms were washed with 1X PBS and scraped off each well and subsequently sonicated and vortexed as previously detailed. Serial dilutions were then plated on selective agar plates.

### Statistics

Differences in bacterial CFUs/mL and CFUs/well between time points or strains were analyzed using one-way ANOVA with Dunnett’s multiple comparison test using GraphPad Prism 6.0 software. To compare the significance between the percentages of bacterial CFUs that remained in the coculture biofilm after gentamicin and ciprofloxacin treatment compared to those calculated in the monoculture biofilms, we used the χ^2^ test^77^.

## Supplementary information

Supplementary Information
