# Supplementary material for: Optimal environmental and culture conditions allow the in vitro coexistence of Pseudomonas aeruginosa and Staphylococcus aureus in stable biofilms
Source: Sci Rep. 2019 Nov 8;9:16284. doi: 10.1038/s41598-019-52726-0 (PMC6841682; doi:10.1038/s41598-019-52726-0)
Supplement: Supplementary file 1 — Supplementary Information [file 41598_2019_52726_MOESM1_ESM.pdf]

Optimal environmental and culture conditions allow the *in vitro* coexistence of *Pseudomonas aeruginosa* and *Staphylococcus aureus* in stable biofilms

Maria del Mar Cendra*, Núria Blanco-Cabra, Lucas Pedraz, Eduard Torrents*

Supplementary Information

Supplementary Fig. S1. pH evolution during monoculture biofilm growth of *P. aeruginosa* and *S. aureus.* pH was measured at 0, 24, 48 and 72 h monoculture biofilm growth in LB, SCFM2, TSB and DMEM medium.

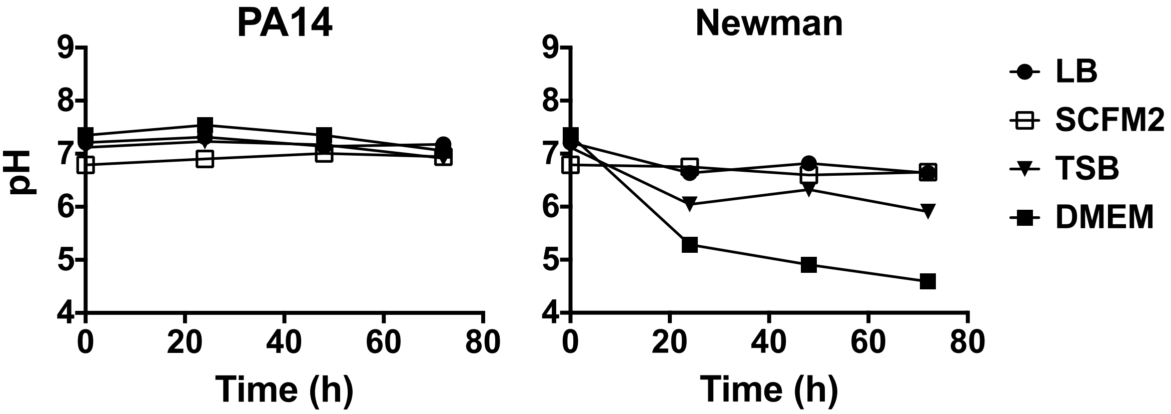

Supplementary Fig. S2. Cristal violet biomass staining of Newman and PA14 mono- and cocultured biofilms formed with DMEM medium on a 96-well plate. The average OD_{570} of 5 wells with the respective error bars indicating the standard error of the mean is shown in the plot. PBS was added as a control.

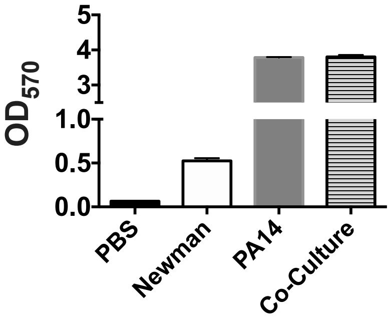

Supplementary Fig. S3. Schematic representation showing how was the oxygen concentration measured in the different static cocultured biofilms. The experiment is set up in a bacterial incubator (**1**) at 37 ºC. The culture grows in a 6-well cell culture plate (**2**) sitting on an EPS box full of moist paper towel (**3**) to keep the system humid. The height of the plate is regulated by a scissor lifting platform (**4**). The oxygen saturation in the system is measured through an OxyMicro device (**5**). The temperature cable (**6**) connects a temperature probe inside the incubator (**7**). An optical fiber cord (**8**) is connected to a syringe-type micro-optode (**9**) ending in a needle (**10**). The needle crosses the lid (**11**) of the culture plate through small holes and the measuring end of the optical fiber (**12**) is immersed in the culture medium (**13**). The OxyMicro processor is connected (**14**) to power and a laptop. The coverslip for cell growth can be placed leaning on a wall for air-liquid interphase measurements (**15**) or fully immersed (**16**). In (a) is shown the complete setting up of the experiment while in (b) are detailed the positions where the oxygen was measured during the co-culture biofilm growth. 6-well plates were purchased sterile, but when required, the system was additionally sterilized using UV light to ensure sterile conditions.

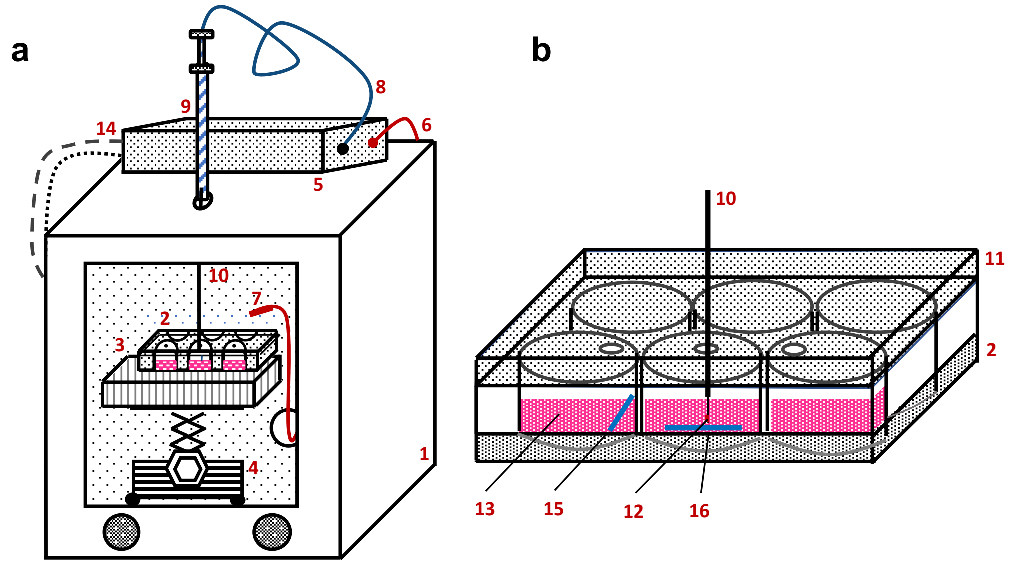

Supplementary Fig. S4. Average population of *S. aureus* Newman microcolonies in a 3-days old continuous flow cocultured biofilm with *P. aeruginosa* PA14. (a) Confocal microscopy micrographs of different Newman's microcolonies grown embedded of *P. aeruginosa* biofilm. Both strains were growing together in continuous flow of DMEM medium for three days. The corresponding average number of Newman cells counted per microcolony is shown in (b).

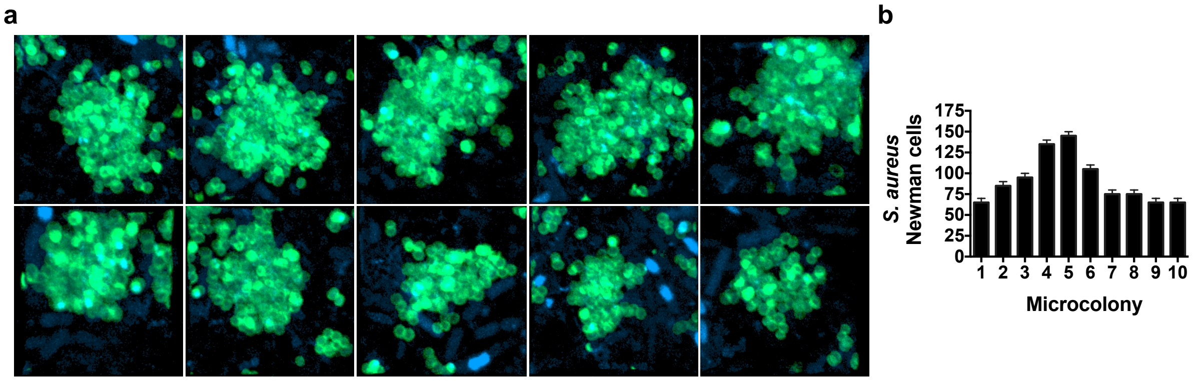

Supplementary Fig. S5. Planktonic *S. aureus* mono-bacterial growth in DMEM and TSB. O/N cultures were washed in 1X PBS, adjusted to initial OD_{550} = 0.05 in DMEM and TSB and inoculated in triplicate in a 96-well polystyrene plate. OD_{550} was subsequently measured every 15 min for 450 min (7.5 hours).

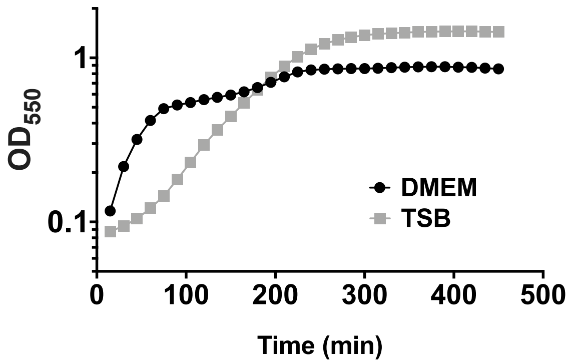

Supplementary Table S1. *P. aeruginosa* PA14 and *S. aureus* Newman cells percentage within the mixed biofilm grown in different media.

	Time (h)	PA14 %	Newman %
**LB**	12	92.95 %	7.04%
	24	99.96 %	0.03 %
	36	99.99 %	0.001 %
	48	100 %	0.00025 %
	60	100 %	0 %
	72	100 %	0 %
**TSB**	12	94.41 %	5.58 %
	24	98.85 %	1.14 %
	36	99.92 %	0.07 %
	48	100 %	0.00001%
	60	100 %	0 %
	72	100 %	0 %
**DMEM**	12	99.57 %	0.42 %
	24	99.96 %	0.039 %
	36	99.98 %	0.011 %
	48	99.99 %	0.008 %
	60	99.99 %	0.0003%
	72	100 %	0 %
**SCFM2**	12	99.00 %	0.99 %
	24	99.97 %	0.022 %
	36	99.99 %	0.0004 %
	48	99.99 %	0.0004%
	60	100 %	0 %
	72	100 %	0 %

Supplementary Table S2. *P. aeruginosa* PA14 and *S. aureus* Newman cells percentage within the mixed biofilm grown in DMEM supplemented with NADPH, BSA, AMP and L-arg at different timepoints.

	Time (h)	PA14 %	Newman %
**DMEM+NADPH**	12	84.16 %	15.84 %
	24	75.33 %	24.66 %
	36	99.81 %	0.18 %
	48	99.98 %	0.016 %
	60	99.99 %	0.0001 %
	72	100 %	0 %
**DMEM+BSA**	12	95.56 %	4.43 %
	24	84.61 %	15.38 %
	36	98.13 %	1.86 %
	48	99.59 %	0.41 %
	60	99.89 %	0.10 %
	72	99.99 %	0.005 %
**DMEM+AMP**	12	91.38 %	8.61 %
	24	88.99 %	11.00 %
	36	99.89 %	0.109 %
	48	99.98 %	0.01 %
	60	99.99 %	0.0001 %
	72	100 %	0 %
**DMEM+L-arg**	12	86.92 %	13.07 %
	24	91.54 %	8.46 %
	36	87.64 %	12.35 %
	48	93.21 %	6.79 %
	60	99.97 %	0.029 %
	72	99.99 %	0.00013 %

Supplementary Table S3. ***P. aeruginosa*** **PA14 and** ***S. aureus*** **Newman remaining biofilm forming CFUs after gentamicin (Gm) and ciprofloxacin (Cpx) treatment of 48 h old mono- and cocultured biofilms.**

Antibiotic	µg/mL	CFU's mean	SD
**Untreated**			
PA14 monoculture	0.00	3.90E+08	1.65E+07
PA14 coculture	0.00	2.83E+08	2.08E+07
Newman monoculture	0.00	1.66E+08	5.29E+06
Newman coculture	0.00	2.31E+06	4.47E+05
**Gm treatment**			
	0.50	3.02E+06	5.90E+05
	1.00	1.03E+06	1.11E+05
PA14 monoculture	2.00	9.77E+04	2.52E+03
	4.00	1.11E+05	2.69E+04
	8.00	1.22E+04	1.65E+03
	0.50	8.37E+07	5.81E+06
	1.00	5.09E+07	1.06E+07
PA14 coculture	2.00	2.78E+07	6.92E+06
	4.00	2.37E+06	2.24E+05
	8.00	5.47E+04	5.03E+03
	0.50	1.14E+06	2.47E+05
	1.00	1.02E+06	1.11E+05
Newman monoculture	2.00	1.69E+04	3.00E+03
	4.00	1.97E+03	4.04E+02
	8.00	1.07E+03	1.53E+02
	0.50	1.64E+07	1.38E+06
	1.00	6.57E+06	4.21E+05
Newman coculture	2.00	2.51E+04	9.02E+02
	4.00	2.08E+03	4.09E+02
	8.00	2.34E+03	2.38E+02
**Cpx treatment**			
	0.50	2.99E+06	8.49E+05
PA14 monoculture	1.00	2.43E+05	2.08E+04
	2.00	1.37E+05	4.04E+04
	4.00	2.68E+04	2.00E+03
	0.50	1.66E+07	1.63E+06
PA14 coculture	1.00	4.90E+06	1.10E+06
	2.00	1.27E+05	3.06E+04
	4.00	3.40E+04	5.29E+03
	0.50	1.82E+06	4.79E+05
Newman monoculture	1.00	2.52E+05	5.33E+04
	2.00	1.15E+05	1.32E+04
	4.00	6.53E+02	1.27E+02
	0.50	1.00E+07	9.08E+05
Newman coculture	1.00	1.62E+06	1.10E+05
	2.00	2.05E+03	2.50E+02
	4.00	4.17E+02	9.07E+01
